# Missed signs of autonomic dysreflexia in a tetraplegic patient after incorrect placement of urethral Foley catheter: a case report

**DOI:** 10.1186/s13037-014-0044-3

**Published:** 2014-11-22

**Authors:** Subramanian Vaidyanathan, Bakul M Soni, Tun Oo, Peter L Hughes, Gurpreet Singh

**Affiliations:** Regional Spinal Injuries Centre, Southport and Formby District General Hospital, Town Lane, Southport PR8 6PN UK; Department of Radiology, Southport and Formby District General Hospital, Town Lane, Southport PR8 6PN UK; Department of Urology, Southport and Formby District General Hospital, Town Lane, Southport PR8 6PN UK

**Keywords:** Spinal cord injury, Autonomic dysreflexia, Tetraplegia, Urethra, Urinary catheter

## Abstract

**Background:**

Autonomic dysreflexia is poorly recognised outside of spinal cord injury centres, and may result in adverse outcomes including mortality from delayed diagnosis and treatment. We present a spinal cord injury patient, who developed autonomic dysreflexia following incorrect placement of urethral Foley catheter. Health professionals failed to recognise signs and symptoms of autonomic dysreflexia as well as its significance in this tetraplegic patient.

**Case presentation:**

A tetraplegic patient started sweating profusely following insertion of a Foley catheter per urethra. The catheter was draining urine; there was no bypassing, no bleeding per urethra, and no haematuria. Patient’s wife, who had been looking after her tetraplegic husband for more than forty years, told the health professionals that the catheter might have been placed incorrectly but her concerns were ignored. Ultrasound scan of urinary tract revealed no urinary calculi, no hydronephrosis. The balloon of Foley catheter was not seen in urinary bladder but this finding was not recognised by radiologist and spinal cord physician. Patient continued to sweat profusely; therefore, CT of pelvis was performed, but there was a delay of ten days. CT revealed the balloon of Foley catheter in the over-stretched prostate-membranous urethra; the tip of catheter was not located within the urinary bladder but was lying distal to bladder neck. Flexible cystoscopy was performed and Foley catheter was inserted into the bladder over a guide wire. The intensity of sweating decreased; noxious stimuli arising from traumatised urethra might take a long while to settle.

**Conclusion:**

Inserting a catheter in a tetraplegic patient should be carried out by a senior health professional, who is familiar with spasm of bladder neck which occurs frequently in tetraplegic patients. Facilities for urgent CT scan should be available to check the position of Foley catheter in spinal cord injury patients when a patient manifests signs and symptoms of autonomic dysreflexia following insertion of a urethral catheter. When an isolated symptom such as flushing or sweating is noticed in a tetraplegic patient, doctors should seek out other signs/symptoms of autonomic dysreflexia.

## Background

Khastgir and associates [1] succinctly summarised the serious medical condition called autonomic dysreflexia. Autonomic dysreflexia is a potentially life-threatening hypertensive medical emergency that occurs most often in spinal cord-injured individuals with spinal lesions at or above the mid-thoracic spinal cord level. Autonomic dysreflexia is poorly recognised outside of spinal cord injury centres, and may result in adverse outcomes including mortality from delayed diagnosis and treatment. Acute autonomic dysreflexia is characterised by severe paroxysmal hypertension associated with throbbing headaches, profuse sweating, nasal stuffiness, flushing of the skin above the level of the lesion, bradycardia, apprehension and anxiety, which is sometimes accompanied by cognitive impairment.

In humans with spinal cord injury, micturition is the most frequent autonomic dysreflexia-inducing stimulus, recurring ~3–6 h daily [2]. Usually, the onset of autonomic dysreflexia is accompanied by symptoms including headache or sweating above the level of injury; however, autonomic dysreflexia also can be “silent” or with minimal symptom such as sweating alone. Symptomatic cardiac arrhythmias can occur in patients, who develop autonomic dysreflexia [3]. In addition to urinary bladder-related causes, autonomic dysreflexia may occur due to syringomyelia, severe faecal loading of colon, manual evacuation of bowels, neuropathic lumbar spondylolisthesis [4], or Charcot’s spine. The latter condition causes headache and sweating of the head associated with trunk movements, such as sitting and turning over in bed, and the movement to perform self-urinary catheterization which causes forced trunk flexion [5]. We present a spinal cord injury patient, who developed clinical features of autonomic dysreflexia following incorrect placement of urethral Foley catheter. Health professionals failed to recognise the signs of autonomic dysreflexia and its significance in this tetraplegic patient.

## Case presentation

A 20-years-old, British, male sustained cervical spinal cord injury (ASIA impairment scale B) at C-5 level in 1970 while teaching gymnastics. He was a physical training instructor in army and he landed on the wrong side of a trampoline. This gentleman had been managing his bladder by penile sheath drainage until 2013 when he underwent surgery for upper gastrointestinal bleeding. Since then, he had been draining urine by urethral Foley catheter.

**Figure 1:**
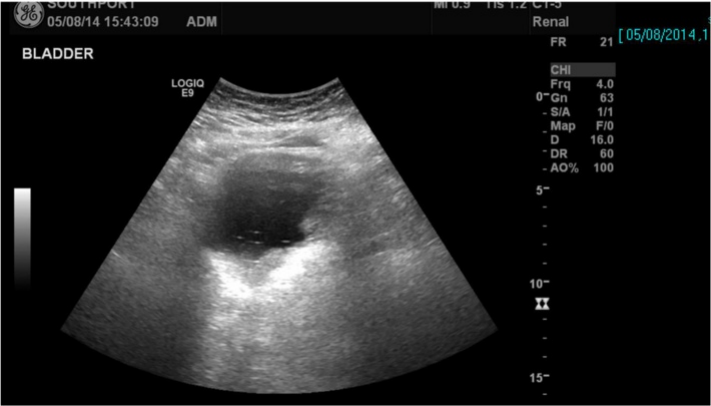
**Ultrasound scan of urinary bladder: the balloon of Foley catheter is not seen, but the importance of this finding was not recognised by radiologist or by spinal cord physician.**

**Figure 2:**
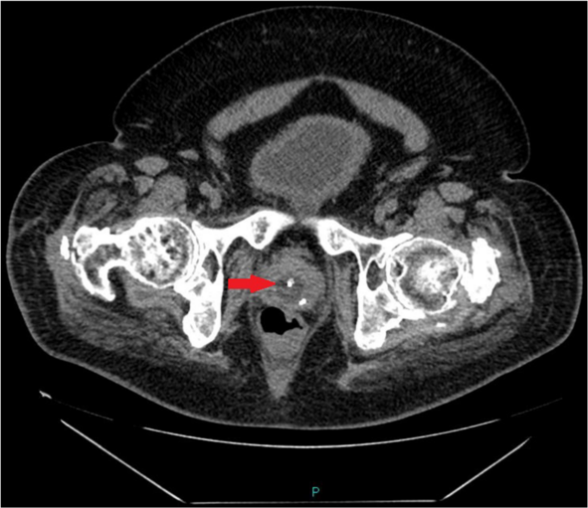
**Axial scan of CT of pelvis performed ten days later: Neither the catheter nor balloon of Foley catheter can be seen inside the urinary bladder.** The balloon of Foley catheter (arrow) is located in dilated prostate-membranous urethra.

**Figure 3:**
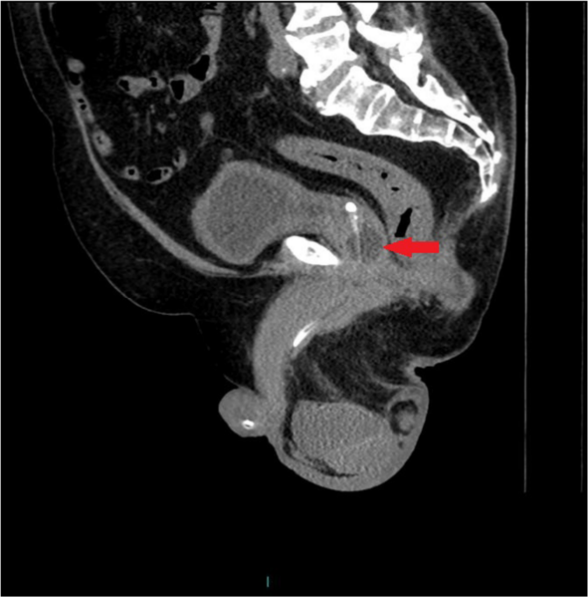
**Sagittal section of CT of pelvis: the balloon of Foley catheter (arrow) is located in dilated prostato-membranous urethra.**

**Figure 4:**
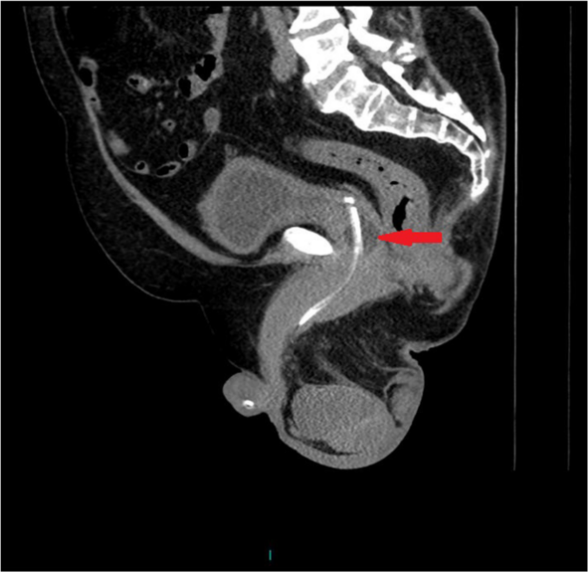
**The tip of Foley catheter, which is radio-opaque, is not lying within the urinary bladder but it is located distal to bladder neck.** The balloon of Foley catheter (arrow) is seen outside the urinary bladder within the urethra.

Following a routine change of urethral catheter by a health professional, this patient started sweating profusely. The Foley catheter was draining urine; there was no bypassing, no bleeding per urethra, and no haematuria. Patient’s wife, who had been looking after her tetraplegic husband for more than forty years, told the health professionals that the catheter might have been placed incorrectly but her concerns were ignored by health professionals. Therefore, this patient came to spinal unit for advice. On clinical examination, this patient was sweating profusely over his head and face. Blood pressure was 140/70 mm Hg. The patient did not have symptoms of urine infection nor did he feel unwell. Ultrasound scan of urinary tract revealed no urinary calculi, no hydronephrosis. The balloon of Foley catheter was not seen in urinary bladder (Figure 1) but this finding was not recognised by radiologist and spinal cord physician. Patient continued to sweat profusely; therefore, Computed Tomography (CT) of pelvis was performed, but there was a delay of ten days. Special attention was taken to include the entire urethra in addition to urinary bladder for scanning, as misplacement of urethral catheter was suspected by the spinal cord physician. CT revealed the balloon of Foley catheter in the over-stretched prostato-membranous urethra (Figures 2 and 3). The tip of Foley catheter was not located within the urinary bladder but was lying distal to bladder neck (Figure 4). Flexible cystoscopy was performed and Foley catheter was inserted into the bladder over a guide wire. The intensity of sweating decreased; noxious stimuli arising from traumatised urethra might take a long while to settle.

**Figure 5:**
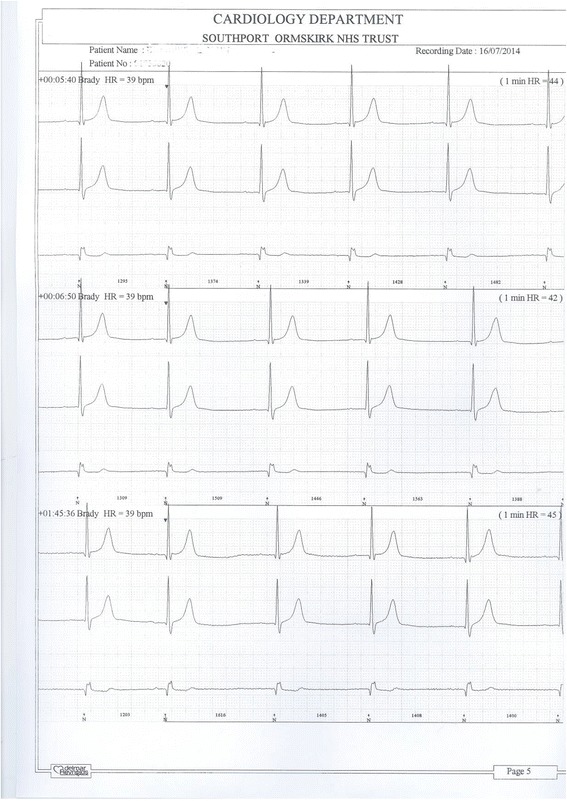
**24-hours ECG shows bradycardia of 39 beats per minute.**

**Figure 6:**
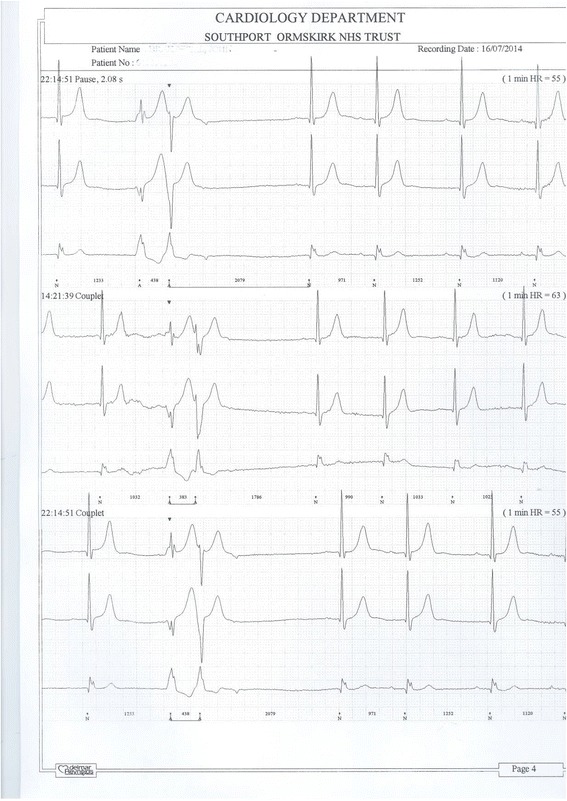
**24-hours ECG shows a pause of 2.08 seconds (topmost recording).**

24-hours ECG monitoring revealed bradycardia: slowest being 39 beats per minute (Figure 5), 214 episodes, and 36 beats at 00:09:04. There was a pause of 2.08 seconds at 22:14:51 (Figure 6). X-ray of pelvis revealed marked osteoarthritic changes in both hips; X-ray of lumbar spine revealed slight scoliosis concave to the right; anterior hyperostosis, most marked at L4/5; disc spacing was well maintained.

Ultrasound scan revealed normal size right kidney measuring 12.5 cm with no hydronephrosis or calculi. The left kidney was atrophic measuring 8.4 cm, cortical depth of 14.1 mm. No calculus was seen. Blood tests: Urea: 5.9 mmol/L; Creatinine: 69 micromol/L; HbA1c: 36 mmol/mol; PSA: 0.15 ug/L. Urine culture yielded growth of >10^8^/L of Staphylococcus aureus. Cytology of urine revealed abundant neutrophils and red cells. Groups of urothelial cells were seen with slightly enlarged nuclei. Some of the groups have a vaguely papillary appearance, which could be seen in low-grade urothelial neoplasms, infection, instrumentation or stones. A follow-up urine cytology revealed a few inflammatory cells and urothelial cells. No malignant cells were seen.

## Discussion

Sweating in a tetraplegic patient can be due to several factors, although dyssynergic voiding, and blocked urinary catheter are common causes. Gorman [6] described a 40-year-old man with a 20-year history of C4 complete tetraplegia, who complained of 5 years of excessive, intermittent, left-sided sweating. The sweating occurred only when the patient was in the seated upright position. There was no associated headache, blurred vision, or blood pressure variability. When examined upright, the patient sweated excessively on the left face and body. When he was laid down, sweating ceased. Skin examination revealed intact ischial regions. Pressure applied to the right ischium for several minutes caused sweating to recur on the left forehead, but it then subsided with release of pressure. This phenomenon was repeatable. Local lidocaine injection in the subcutaneous tissues around the right ischium and subsequent use of lidocaine transdermal patches halted the contralateral sweating in the upright position. Pressure mapping analysis showed increased pressure in the region of the right ischial tuberosity. The patient’s gel cushion was replaced with an air-filled cushion, providing significant ongoing relief from the hyperhidrosis. In our patient, X-ray of hip joints revealed degenerative changes, which were more marked on left side. We advised our patient to get pressure mapping while seated on his chair so that a greater pressure-relieving cushion could be provided, if required. When the balloon of Foley catheter was found to be located in the urethra, this patient was advised not to sit up; sitting on the inflated balloon can predispose to pressure ulceration of perineal skin.

When an isolated symptom such as flushing or sweating is recognized in a tetraplegic patient, it is important to seek out other signs and symptoms of autonomic dysreflexia. Health professionals should bear in mind that sweating is not the only sign of autonomic dysreflexia. In this patient, bradycardia and systolic blood pressure of 140 mm Hg, were noted when he was examined in spinal injuries unit. Tetraplegic patients typically have baseline systolic blood pressure of 90 – 110 mm Hg. Autonomic dysreflexia is defined as blood pressure elevation to >30 mm Hg above baseline.

Profuse sweating following insertion of urethral catheter in the present case should have alerted health professionals to check the position of Foley catheter by appropriate imaging techniques. Had ultrasound scan or CT been done promptly, the medical mishap of incorrect placement of Foley catheter would have been detected without delay. Autonomic dysreflexia can become a life-threatening event in tetraplegic subjects; emergency room physicians should be aware of the varied clinical features of autonomic dysreflexia, and take prompt action in order to avert serious complications such as convulsions [7], brain haemorrhage [8], and death [9].

## Conclusions and take-home message

1. Inserting a catheter in a tetraplegic patient should be carried out by a senior health professional, who is familiar with spasm of bladder neck which occurs frequently in tetraplegic patients.
2. Health professionals should listen to spinal cord injury patient and his/her relative/carer. When the patient started sweating following insertion of the catheter, patient’s wife, who had been looking after her tetraplegic husband for more than forty years, said that the catheter was not in proper place. But the health professionals did not take her remarks seriously.
3. Both the radiologist and spinal cord physician made a mistake as well. During the initial ultrasound scan, the balloon of Foley catheter was not seen inside the urinary bladder. But the catheter was draining urine; there was no bypassing. Both radiologist and spinal cord physician were in a hurry to see the long list of patients. They did not have time to pause, think and reflect upon the complex clinical situation.
4. Organisationally, more time should be allotted for spinal cord injury patients, who present with complex clinical problems.
5. Furthermore, facilities for urgent CT scan should be made available round the clock to check the position of Foley catheter in spinal cord injury patients when in doubt and especially, when a patient manifests signs and symptoms of autonomic dysreflexia such as sweating, bradycardia, or increase in blood pressure, following insertion of a urethral catheter.
6. When the balloon of Foley catheter is located in the perineum, a spinal cord injury patient should not sit up; sitting on the inflated balloon will predispose to pressure ulceration of perineal skin.

## Consent

Written informed consent was obtained from this tetraplegic patient, for publication of this Case report and accompanying images. A copy of the written consent is available for review by the Editor-in-Chief of this journal.
